# Buffalo Medical College

**Published:** 1848-12

**Authors:** 


*Buffalo Medical College.*—The annual session in this Institution commences on this day, Wednesday, Nov. 29. From the number of students already assembled, the class may be expected to exhibit a considerable increase over the attendance at the last session, notwithstanding the five months' course, and a repudiation of the credit system *without a single exception.* About forty were in attendance at the preliminary dissecting term.

